# Supplementary material for: Could fish aggregation at ocean aquaculture augment wild populations and local fisheries?
Source: PLoS One. 2024 Apr 17;19(4):e0298464. doi: 10.1371/journal.pone.0298464 (PMC11023196; doi:10.1371/journal.pone.0298464)

Fish aggregation at ocean aquaculture can augment wild populations and local fishing

Supplemental Information

Jessica L. Couture, Darcy Bradley, Benjamin S. Halpern, Steven D. Gaines

This document includes the supplemental information for "Fish aggregation at ocean aquaculture can augment wild populations and local fisheries".

Figure S1

Equilibrium biomass relative to total biomass with no farm, for different levels of attraction to farms, fishery management, and varying total area coverage. For (A, C) one large contiguous farm and (B, D) total farm area broken up into smaller separate farms. Top plots (A, B) are run under strong fisheries management, bottom plots (C, D) are under weak management. Biomass difference is calculated as the difference between catch biomass with the farm scenario and a scenario with no farm.

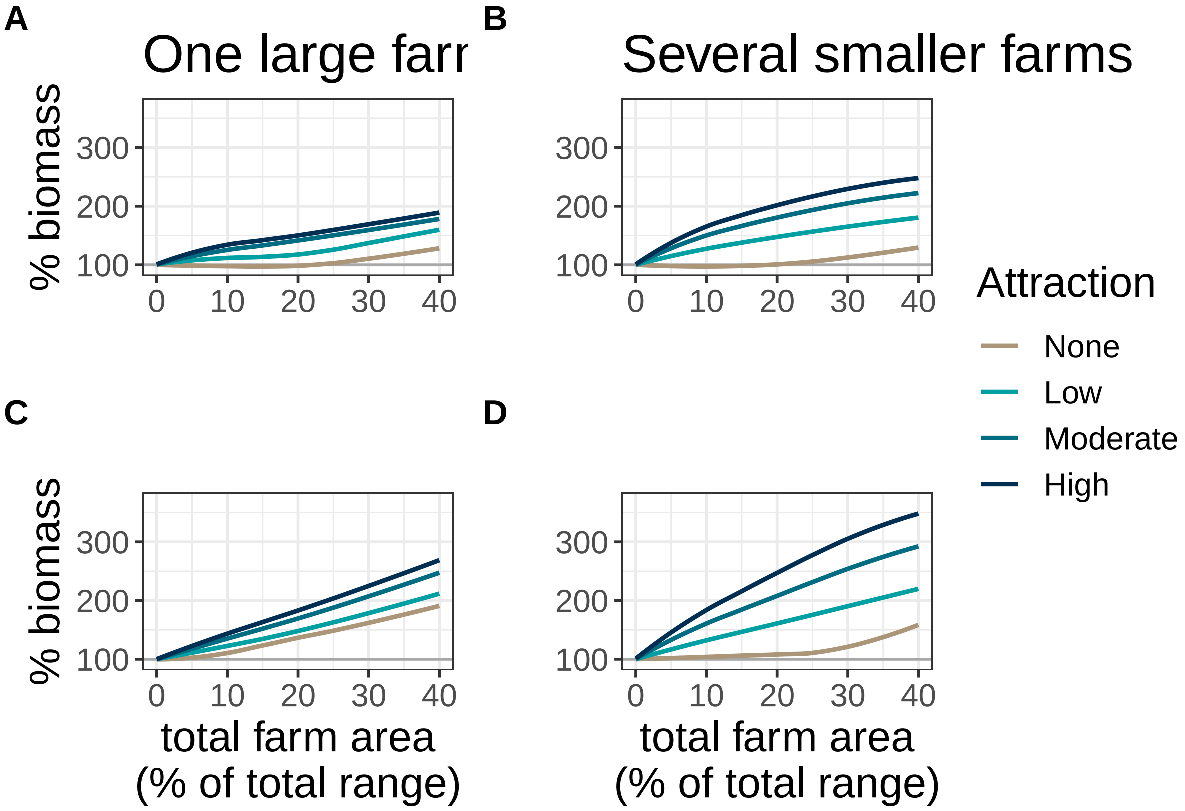

Figure S2

Farm impacts over time for a **high value open access fishery**. In each plot the level of attraction is varied. Top plots represent one large farm at 20% total coverage (A, B), and the bottom plots represent the 20% farm area broken into several smaller farms (C, D). Biomass and catches are in relative to the base scenario with no farm (farm scenario/no farm scenario).

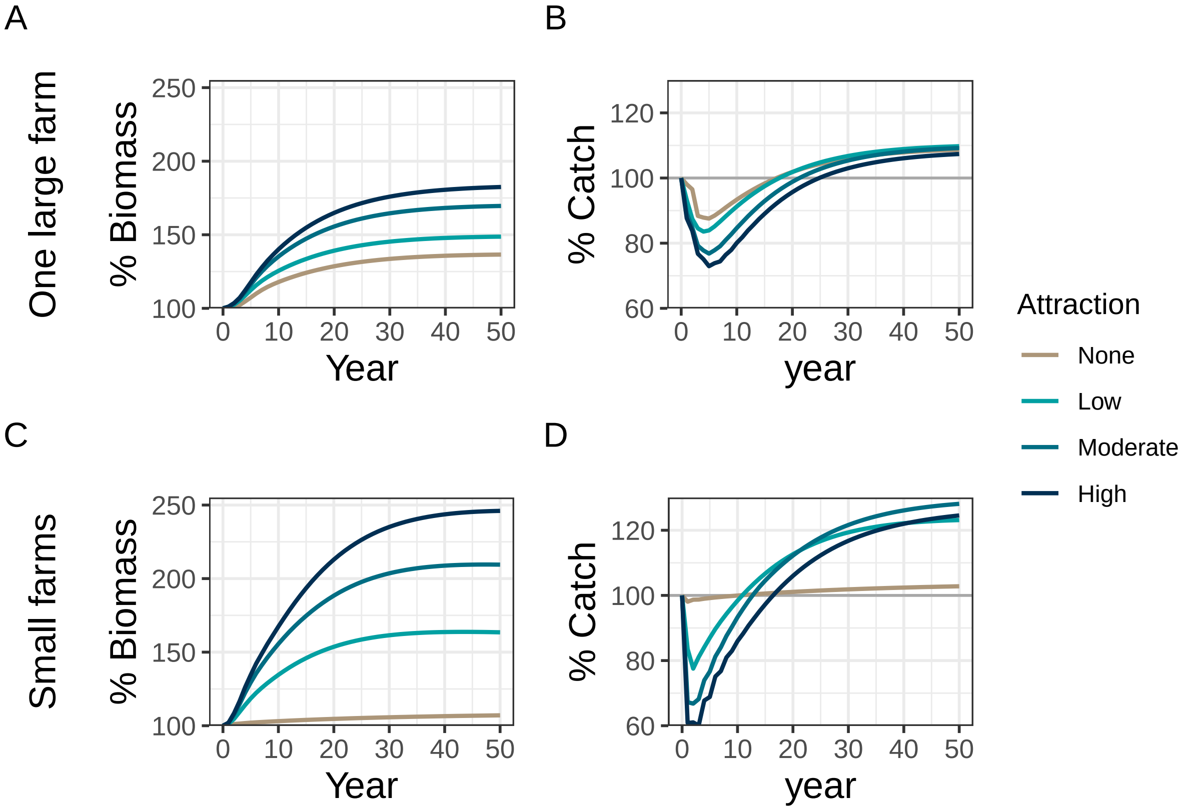

Figure S3

Farm impacts over time for a fishery fished at constant effort at maximum sustainable yield. In each plot the level of attraction is varied. Top plots represent one large farm at 20% total coverage (A, B), and the bottom plots represent the 20% farm area broken into several smaller farms (C, D). Biomass and catches are relative to the base scenario with no farm (farm scenario/no farm scenario).

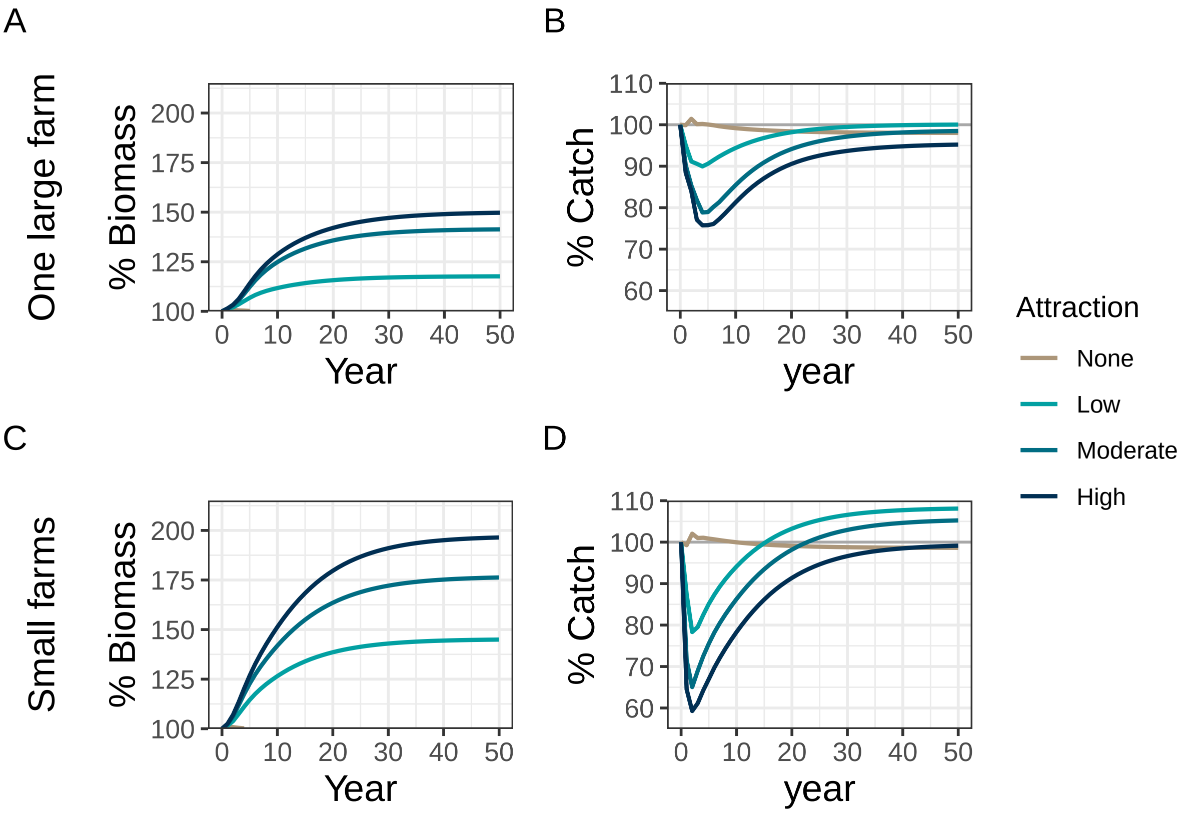

Figure S4

Equilibrium differences in total biomass and catch biomass for different species based on adult movement for farm scenarios with very damaging effects on the wild population (damage > fishing mortality). Different farm designs are tested by farm size resulting in the indicated number of farms.

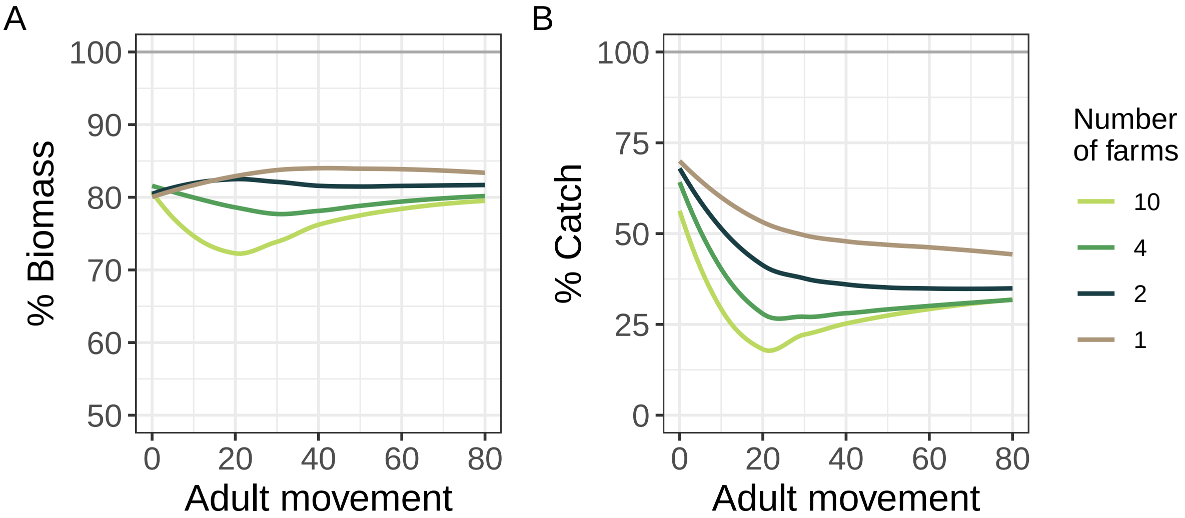

Figure S5

Relative differences in total biomass compared to no farm over a range of total farm areas. Farms are all divided into several smaller farms. Farm scenarios with positive (A, C) and negative impacts to the wild population (B, D), under strong (A, B) and weak (C, D) fishery management.

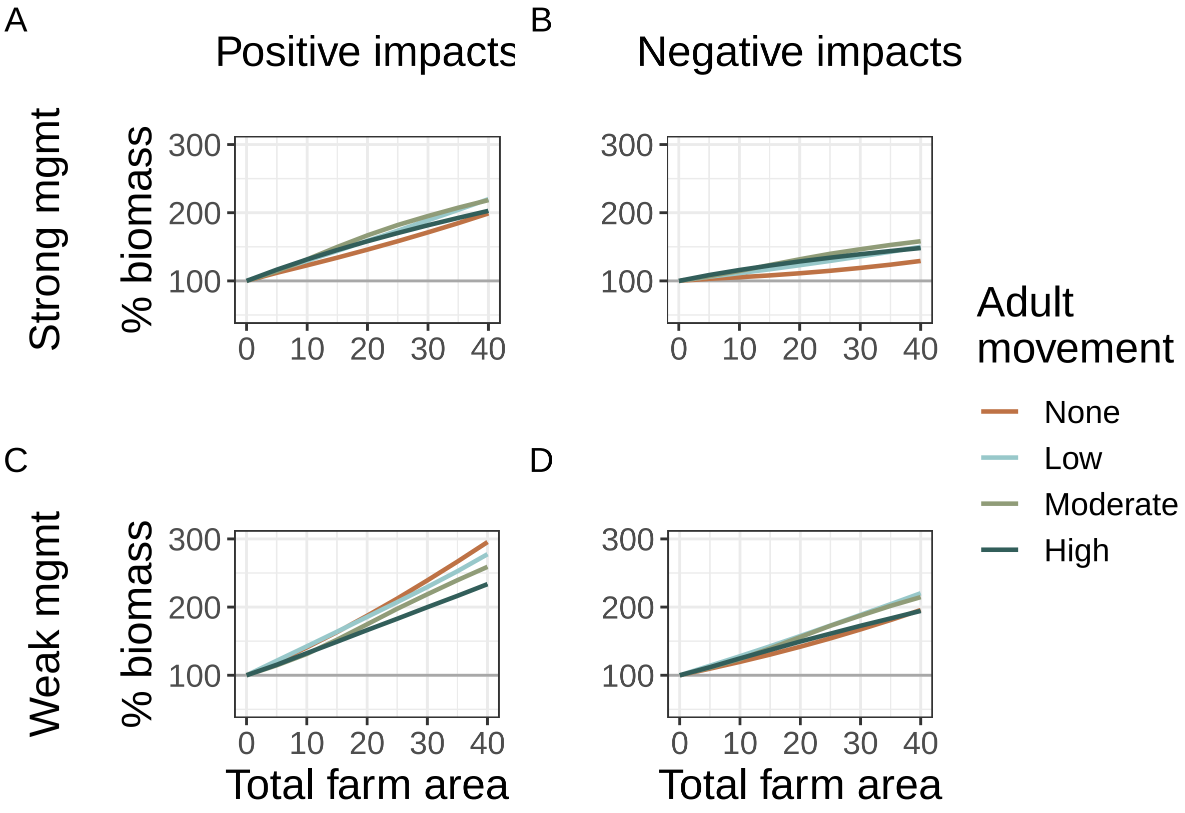

Figure S6

Relative differences in total biomass (A, C) and catches (B, D) under strong (A, B) and weak (C, D) fishery management compared to no farm over a range of total farm areas. Farms are all divided into several smaller farms. Here farms have varying impacts on wild population natural mortality rates (0-20% negative impacts).

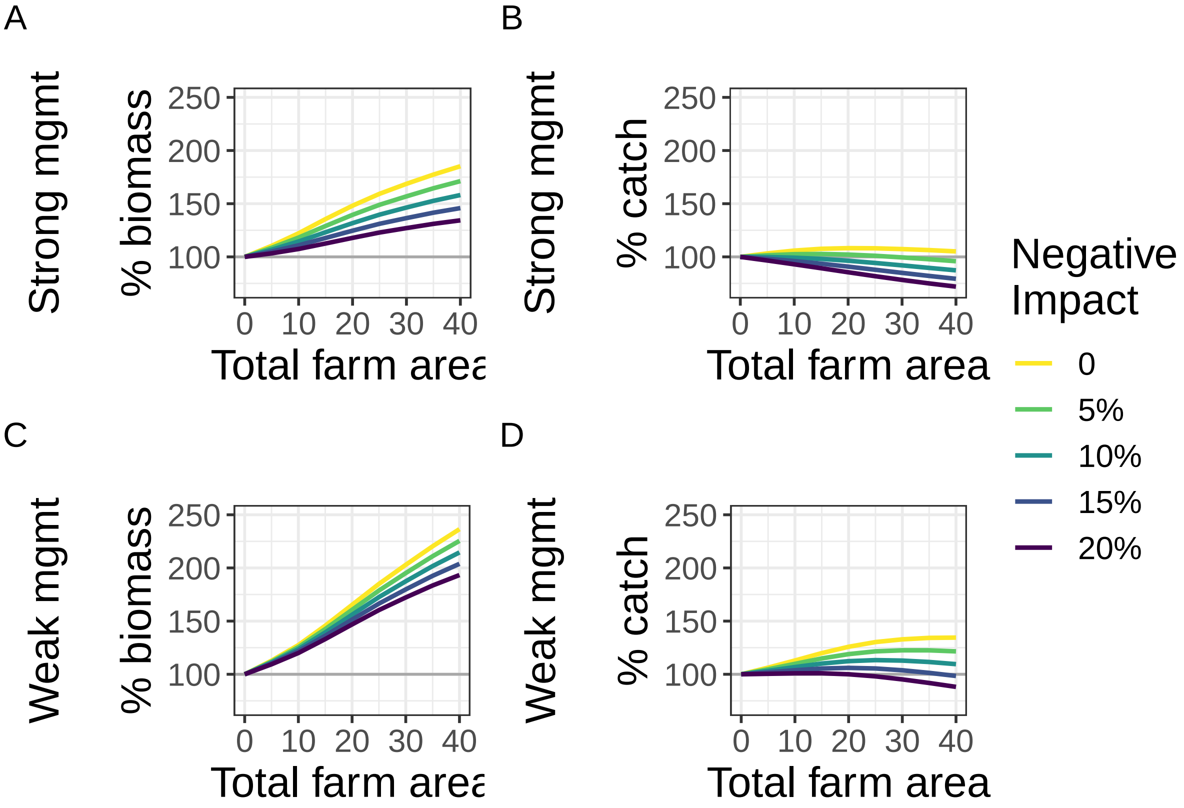

Supplement: S1 File — (PDF) [file pone.0298464.s001.pdf]
