# Supplementary material for: Could fish aggregation at ocean aquaculture augment wild populations and local fisheries?
Source: PLoS One. 2024 Apr 17;19(4):e0298464. doi: 10.1371/journal.pone.0298464 (PMC11023196; doi:10.1371/journal.pone.0298464)
Supplement: Attachment — Submitted filename: FisheriesModel-Review-23-28349.docx [file pone.0298464.s002.docx]

Review of “Fish Aggregation at Ocean Aquaculture Can Augment Wild Populations and Local Fisheries”

The authors attempt to tackle the pressing issue of how an increasing aquaculture and fish farming industry may affect existing fisheries and wild target fish populations. The authors utilize robust modeling techniques to assess how catch and biomass would change based on various situations such as: large farms vs. many smaller farms, positive impacts via aggregating at farms vs. negative impacts via aggregating at farms, and various levels of both movement and attraction to the fads. The authors conclude that farms generally have a positive impact and can act as a refuge for fish to restock overfished populations. As a result, intelligent farm design can help increase catch for commercial fisheries and increase wild stocks. However, they state that more empirical information about fish behavior in regard to migration to, residency, and emigration away from fish farms as well as the direct impacts, whether net positive or negative, is needed for most fish species that may potentially interact with fish farms.

I would like to commend the authors for their submission as a lot of time and work went into the creation of these models, and a they show lot of attention to detail in how and what to account for within the model. However, I cannot recommend this paper for publication without major revisions. The authors are attempting to assess every and all situations/species within one paper, while also making strong conclusions based on completely simulated data. By assessing every species that may interact with fish farms, they render the model ecologically impossible and thus detract from their conclusions. For instance, pelagic fish do not aggregate at FADs long enough for them to provide anything close to a spillover effect on wild populations. Furthermore, the scale of farm coverage is ecologically impractical for many species. There are not many species in which you could cover 40% of their home ranges or occupied space with farms, whether those are spread out or a single farm (Fig 2). Because of this, the conclusions are more or less ecologically meaningless to most pelagic species. Lastly, as far as I can tell, there is no real data integrated into the model, whether it’s life history data or stock population data. Yet, the authors make strong conclusions on the impact on farms from completely simulated, abstract data. There is of course merit in modeling data-limited fields such as this one to assess possible outcomes, but the authors’ implications for the results of this paper extrapolated too far based on the real-world applicability of this study. In essence, this manuscript straddles the line between a well-thought-out, well-defined theoretical model, and a model with no validation that is only loosely applicable. It would suit the authors to choose which direction they would like to take the paper to streamline it.

I would suggest the authors make one of the following possible revisions to the paper. 1.) Vastly tone down any implications/conclusions from the manuscript and present the model as purely theoretical in its entirety for use in fisheries monitoring when data become more readily available in the future. This is the easiest, as the code is freely available at the time of reviewing this manuscript. 2.) Specify an ecological system with which you model the data or even a specific species. For example, only include pelagic species, or only include nearshore species. This will allow you to make the model more specific, and potentially more applicable. 3.) Validate this model with data from a fishery with known data to see how it performs with real data input as a case study for the model as presented.

The authors are of course, free to revise as they wish. However, this manuscript presents a model that is far too broad to make the statements found in the results and discussion. Below are specific points that should be addressed to increase the strength of the manuscript.

Line 63-67. This is a run-on, I would split this up.

Line 88-92. This is another run-on.

Line 88-98. Maybe I missed it, but is there any evidence that fish farms act as FADs? The only papers cited here don’t specify fish farms as FADs in any way. While I generally agree with you, attraction is a major variable within the model presented. I would add at least a couple citations backing up this statement.

Line 99-108. This is my biggest discrepancy with the authors. While yes, MPAs serve as stock rebuilding functions, is there any actual evidence that fish farms specifically act as MPAs? If they do act as MPAs, is there any evidence that they are effective? Besides the fact that farms physically restrict fishing, MPAs largely function because of strategic placement. They are used to protect a large breeding population, specific vulnerable life history stages, breeding grounds, entire ecosystems etc. Is the argument that a random fish farm in the middle of the ocean does the same thing? Does this only apply to fish farms that are specifically within MPAs already? Is the spillover argument that the fish in the farm are producing gametes that are fertilized by other farmed animals or the wild populations to increase wild populations? This is unclear and not very well supported within the text. Literature cited is related to marine reserves, but nothing is cited for fish farms acting as MPAs. While this is going into the weeds a little bit, this is a foundational assumption within the model. This needs to be well cited or have data provided very clearly otherwise it undermines the conclusions based on the model.

Line 135-140. Run-on sentence, split up. Also, it needs to be specified which values for the fish life history were used (growth, reproduction, etc), and I would put them here. If they were pulled from Ovando et al., 2016, specify which species. Again, weeds, but these are important variables.

Line 148. Very well-defined variables in this and the next section that make it clear what is being used in the model.

Line 150-153. This is a major point of contention within the model. After a brief look at Ovando et al.’s 2016 paper, they used a 1-dimensional model to represent fish dispersal around an island, correct? Is this a model framework that theoretically aligns with such a broad model as described in this manuscript? This model should make the same assumptions that the original paper did, and limit the conclusive statements provided to that situation, or alter the model framework to reflect a more general situation.

Line 172-174. These definitions make sense, but what are the actual inputs for the model, and how do they differ?

Line 200-202. Citation?

Line 206-207. This is why it is so important to provide evidence that fish farms are actively playing an MPA role and not just a FAD role. This is a major factor within the model that is largely unsupported.

Line 226-228. Why 10%? Is there any reason for this number or is 10% largely arbitrary?

Line 229. I think you should add all of the figures in the supplementary information to the main text. It’s better to show the results of the model instead of just talk about them.

Line 237-239. I think these are incredibly useful metrics being presented here. However, is even a 10% area realistic simply due to farming? These are farms that are ~1 square km, as cited in this manuscript. More evidence that the scope of this manuscript might need to be narrowed to make the results more believable.

Line 256. Sessile is not a life history pattern observed in fish. While this is a concise term within the model to indicate no movement out of the farm once settled, it does not describe fish ecology.

Line 297-303. I possibly missed it, but is there any graphical representation of the biomass under positive impacts? They are discussed in this paragraph but no figure is cited.

Line 323-340. I appreciate the author’s observations about the limitations of the model being presented. However, without providing any real-world data or evidence, why should the readers believe any of the results of this model can be applied to real-world ecosystems?

Line 347-350. Wait, isn’t the argument that fish farms reduce fishing pressure due to inaccessibility?

Line 350-352. There are plenty of FADs that exist at the moment. Is there any evidence that large FADs interrupt migration patterns right now?

Line 366. Have real species behaviors been included in the model? Or are these abstract representations of potential behaviors? A nitpick, but very important to the interpretation of results.

Line 375-376. I would remove this sentence or rephrase this. Would new data then be incorporated into the model presented, or should management be based solely on empirical data?

Line 410-412. This is why validation specifically from the authors is sorely needed. Presenting a model without validation reduces the usability of the model.

Line 417-420. This begs the question of why commonly available parameters weren’t included in the model in the first place. Wouldn’t that provide a stronger case for using this model?

Line 423-424. Case in point for why conclusions and conclusive language need to be softened within the manuscript.

Line 429-431. The range of species here all have different very different levels of movement, dispersal, age/length at maturation, and fecundity. Won’t these need to be modeled differently to account for the disparity in all of these variables?

Line 431-435. It’s not quite clear why these species are included here. None of these organisms, which again have very different life histories and dispersal methods, were included in the model. Won’t these species need a completely different model to get accurate results from simulated situations?

Line 455-457. Is there any evidence that this is true? Not all fish species are attracted to FADs.

Again, I would like to commend the authors on their contribution to the field. I think this is a timely, and important piece of work. However, there is too much of a discrepancy between the range of species abstractly included in the model and the actual ecology of these species.
