# Supplementary material for: Could fish aggregation at ocean aquaculture augment wild populations and local fisheries?
Source: PLoS One. 2024 Apr 17;19(4):e0298464. doi: 10.1371/journal.pone.0298464 (PMC11023196; doi:10.1371/journal.pone.0298464)
Supplement: Attachment — Submitted filename: PONE-28349-R1-Rex-Answer.docx [file pone.0298464.s004.docx]

I want to thank the authors for addressing all of my concerns succinctly and clearly. The reduction of hard conclusions has done a lot to present this model as purely theoretical. I recognize the difficulty in testing these results in real-world situations and thus the need for theoretical modeling to understand the potential effects of these management decisions. The manuscript, as is, presents potentially testable hypotheses for future work to explore. However, without the presentation of a case study, I do wonder if the broad nature of this model will prevent it back from proper predictions of real-world ecology. With that said the authors have done enough to properly answer my concerns and preface their previously hard conclusions with proper ambiguity. I can recommend this manuscript for publication.
